# Rhabdomyolysis in a Patient With a Possible Mitochondrial Pathogenic Variant in the Peri-operative Period: A Case Report

**DOI:** 10.7759/cureus.73577

**Published:** 2024-11-13

**Authors:** Joyce Kam, Rabiu Momoh

**Affiliations:** 1 Critical Care Medicine, Medway Maritime Hospital, Gillingham, GBR

**Keywords:** compartment syndrome, creatine kinase, dialysis, genetic disorders, hyperkalemia, m.9176t>c (atp6) mitochondrial pathogenic variant, maternal inheritance, mitochondrial disorder, renal failure, rhabdomyolysis

## Abstract

Mitochondrial disorders are often underrecognized as potential causes of rhabdomyolysis, a condition characterized by acute muscle breakdown that can lead to local and potentially systemic complications, with the possibility of being life-threatening. Accounts of rhabdomyolysis as a peri-operative complication associated with mitochondrial disorders are rare; therefore, this study is noteworthy. We describe a case of rhabdomyolysis that occurred during the peri-operative period in a middle-aged male with Charcot-Marie-Tooth (CMT) disease-like peripheral neuropathy. Importantly, genetic studies confirmed that the patient's mother, sister, and maternal uncle carried the m.9176T>C (ATP6) mitochondrial pathogenic variant, which follows a maternal inheritance pattern. This suggests that the patient may have inherited the disorder as well.

## Introduction

Rhabdomyolysis is the rapid dissolution of damaged skeletal muscle [1]. Patients’ presentation may vary from being asymptomatic to experiencing muscle fatigue to electrolyte imbalance and acute kidney injuries. Fifteen percent of acute kidney injuries are thought to be caused by rhabdomyolysis [1]. Rhabdomyolysis causes a rise in myoglobin, aldolase, and lactate dehydrogenase, as well as electrolyte imbalance and the release of creatine kinase, which is the most sensitive laboratory test. The most common causes are traumatic injury, drugs, toxins, and infections and less frequently manifested as part of mitochondrial diseases.

Mitochondrial diseases are often referred to as genetic defects which have a downstream effect on oxidative phosphorylation which impairs ATP production. They can also cause dysfunction of beta-oxidation of long-chain fatty acids in the mitochondria [2]. Precautions are recommended when treating patients with mitochondrial disease in the peri-operative period to reduce metabolic burden and prevent complications, such as respiratory failure, cardiac depression, and rhabdomyolysis [3].

In this study, we present a case of a middle-aged man with a Charcot-Marie-Tooth (CMT)-like peripheral neuropathy in whom a probable genetic mutation was suspected, as the patient's mother, sister, and maternal uncle were confirmed to have m.9176T>C (ATP6) mitochondrial pathogenic variant on genetic studies done on them. He presented with rhabdomyolysis requiring renal replacement therapy in the intensive care unit, following an osteotomy procedure. Although patients with CMT disease may experience episodic rhabdomyolysis, we have reviewed the impact of the surgical or peri-operative period in exacerbating or triggering this manifestation in patients with MT-ATP611 gene mutation and CMT-like peripheral neuropathy.

## Case presentation

A 46-year-old male patient presented to the hospital with generalized malaise and bilateral lower limb pain which he noted following a collapse in his home. He awoke from this collapse without any intervention after about 2 h and found himself on his couch. He reported no head trauma, no history of seizures, and noted no recent unwellness or recent infections. No previous episode of a similar collapse episode was reported. He had, however, undergone an osteotomy of the second right proximal interphalangeal joint three weeks prior for deformities resulting from peripheral sensory and motor neuropathy. The peripheral neuropathy started after 40 years of age in the patient. He reported to have been recovering well at home following the procedure and his surgical wound site had healed. He reported no fever, chills, or rigors, he had no change in his normal bowel habits but noted a reduction in his urine output and darkened urine afterward.

**Figure 1:**
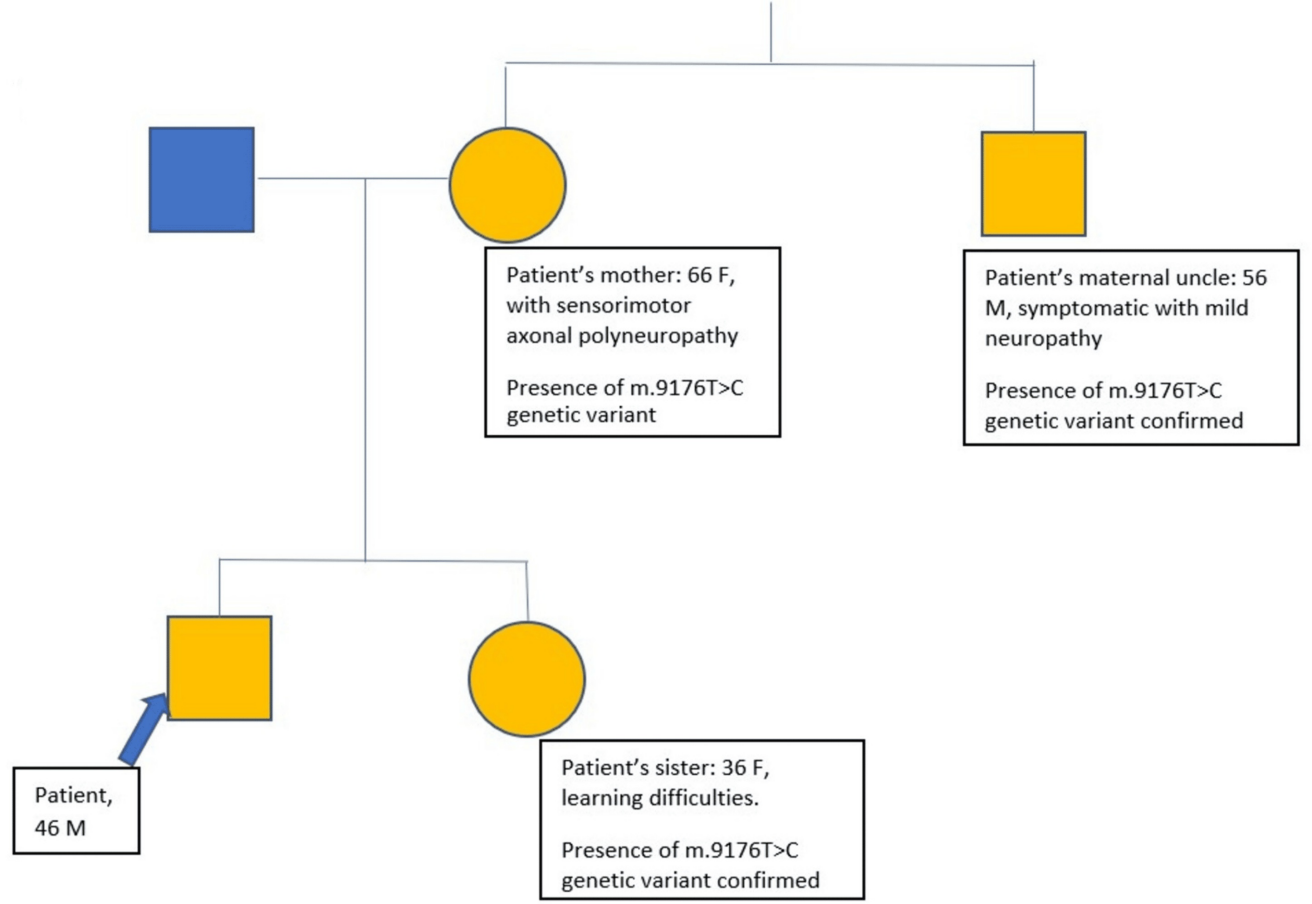
Family tree depicting the inheritance of patient's family line with m.9176T>C mutation. The yellow shape fill depicts possible or confirmed involvement of the m.9176T>C mutation.

The patient's mother, sister, and maternal uncle were known with a formal genetic study diagnosis of m.9176T>C (ATP6) mitochondrial pathogenic variants (family tree is presented in Figure 1). The patient's maternal uncle had a mutation present at a heteroplasmy level of 91% in the blood and 96% in the urine sample studied. The patient, his mother, and his maternal uncle were affected by either mild or severe form of peripheral neuropathy. The patient's mother was on regular treatment with oral gabapentin 300 mg TDS, oral primidone 50 mg OD, coenzyme Q, multivitamins, calcium supplement, and cod liver oil for the care of her sensorimotor axonal polyneuropathy. The decision to undertake genetic screening for the patient's family was made at the suggestion of the family physician of the patient's granddaughter (for health reasons not available at the time of this report). Notably, two years prior to this report, following an orthopedic procedure (aided with tourniquet use) related to his CMT-like peripheral neuropathy, the patient required intensive care unit admission for rhabdomyolysis, necessitating renal replacement therapy.

**Figure 2:**
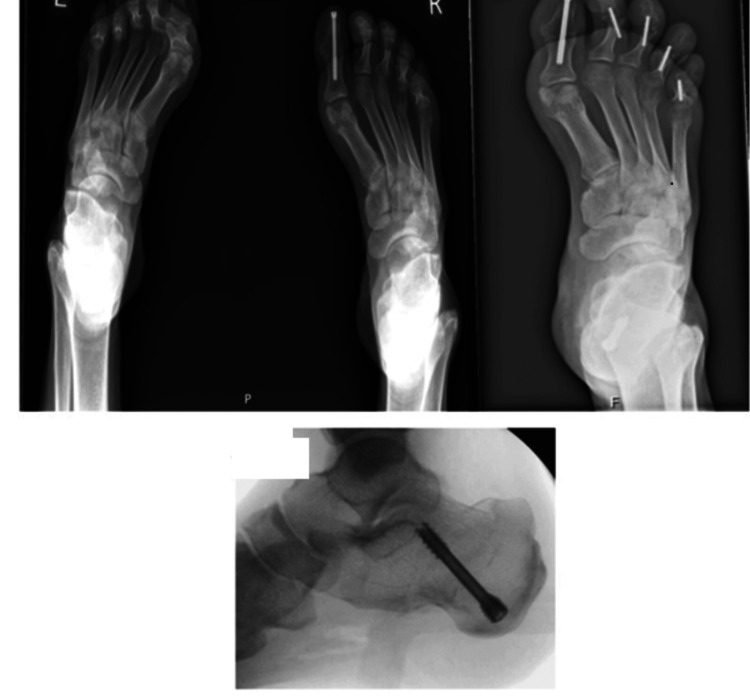
Evidence of instrumentations done on patient's right foot.

Physical examination revealed a drowsy patient at admission, with a Glasgow Coma Scale score of 13 (E3V4M6). He required supplemental oxygen at a rate of 2 L/min via nasal cannula to maintain oxygen saturation levels above 94%. He presented in a hypotensive state, and a urethral catheter was draining dark-colored urine. Significant tenderness was noted bilaterally in both the calf and thigh regions, with swelling more pronounced on the left side. Neurological assessment of the lower limbs was limited due to pain; however, the patient exhibited evident bilateral foot drop, positive plantar reflexes, and reduced tactile sensation in the right foot. The surgical site on the right second toe had healed appropriately, displaying no signs of infection. The evidence of instrumentations done on the patient's right foot is presented in Figure 2.

**Figure 3:**
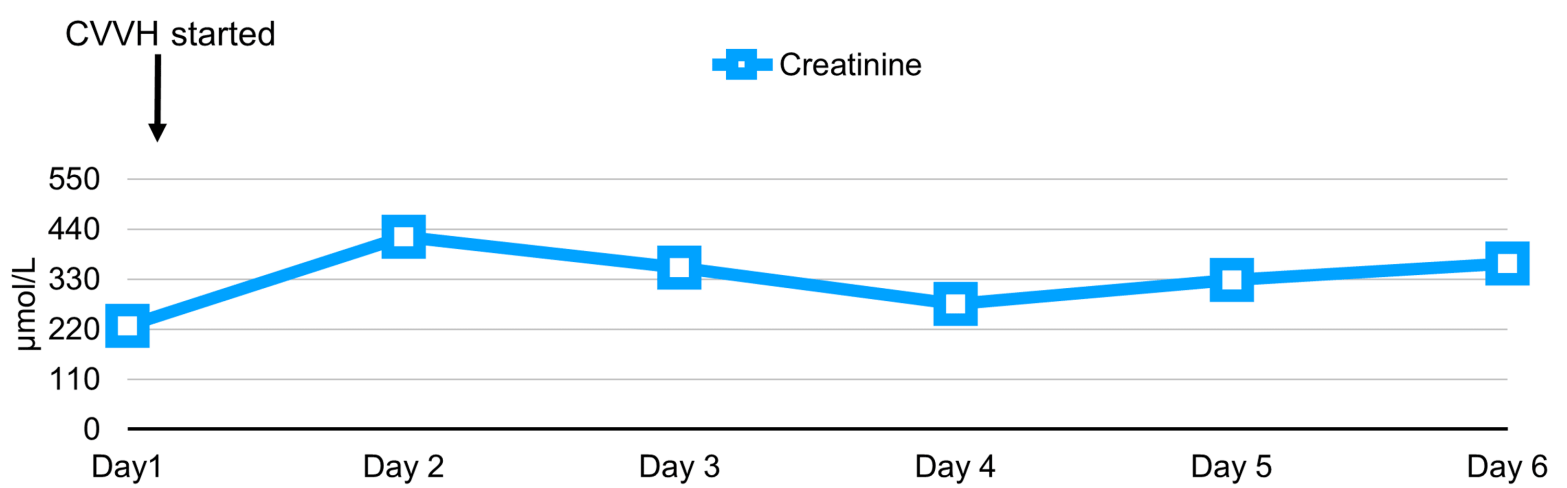
Trend of serial serum creatinine studies done on patient with initiation of CVVH. CVVH: continuous veno-venous hemodiafiltration

**Figure 4:**
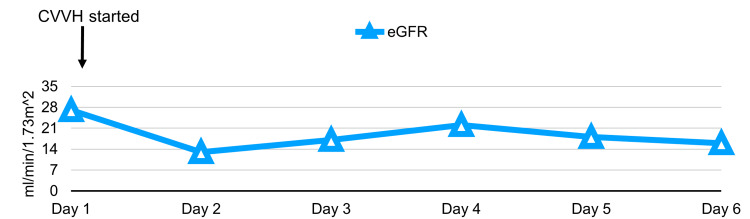
Serial eGFR trend in patient. eGFR: estimated glomerular filtration rate; CVVH: continuous veno-venous hemodiafiltration

**Figure 5:**
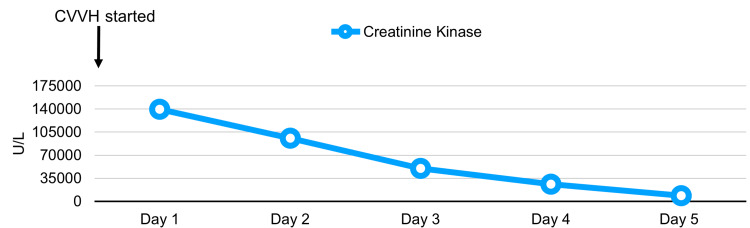
Serial serum creatine kinase study results. CVVH: continuous veno-venous hemodiafiltration

**Figure 6:**
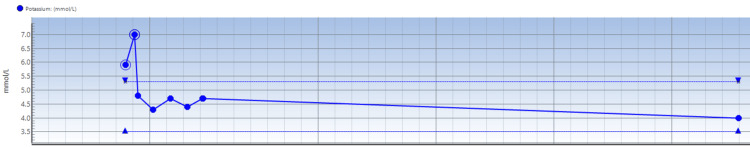
Trends from serial serum potassium studies. Note hyperkalemia resolved with hemodiafiltration and other medical treatments.

The following laboratory results indicated stage 2 acute kidney injury: creatinine level of 227 µmol/L (Figure 3), estimated glomerular filtration rate of 27 mL/min/1.73 m^2^ ​​​​​​and rhabdomyolysis (Figure 4), with a creatine kinase level recorded at 139280 U/L (Figure 5). Initial potassium levels were elevated at 5.9 mmol/L (Figure 6).

**Figure 7:**
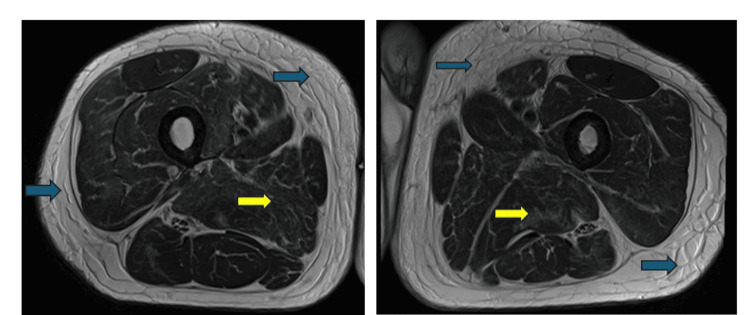
Transverse MRI section through both thighs revealed subcutaneous tissue edema (blue arrows) and edematous changes affecting multiple muscle compartments (yellow arrows).

**Figure 8:**
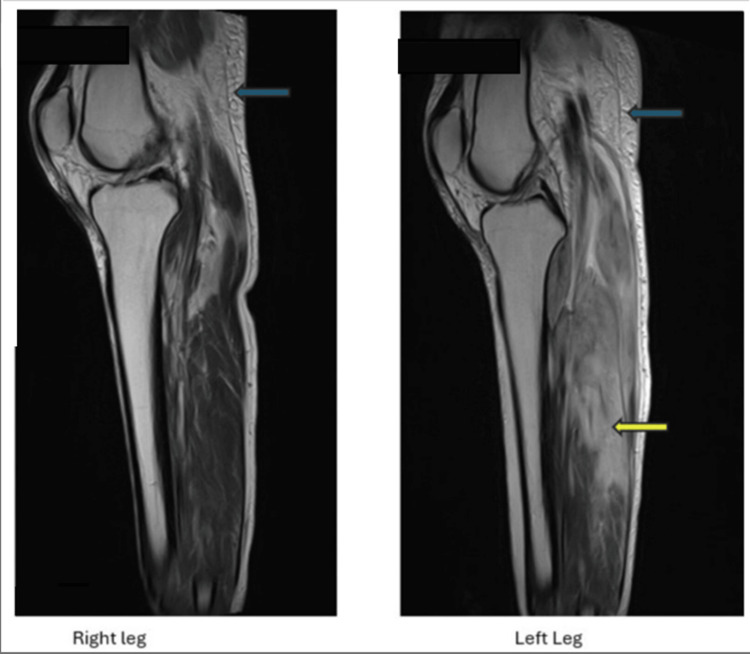
Sagittal MRI sections of both legs showing edematous subcutaneous tissues (blue) and muscle groups (yellow).

Other significant blood test results included a raised white blood cell count of 19.4 × 10^9^/L and a D-dimer level of 678 mg/L. A computed tomography (CT) scan of the lower legs revealed diffusely hypodense and enlarged gastrocnemius muscles bilaterally, more severe on the left side, as well as bilateral involvement of the soleus muscles. Intermuscular fluid was observed bilaterally, accompanied by subcutaneous and intermuscular fat stranding. Subsequent magnetic resonance imaging (MRI) of the lower limbs demonstrated bilateral patchy edematous changes affecting multiple muscle compartments (Figures 7, 8). A Doppler venogram study of the lower limbs excluded the presence of deep vein thrombosis in both lower extremities. Additionally, a CT scan of the head showed no evidence of acute intracranial pathology.

The patient was transferred to the intensive care unit for further care from the emergency department. He was established on continuous veno-venous hemodiafiltration (CVVH) due to rhabdomyolysis and acute kidney injury with anuria. He was commenced on intravenous flucloxacillin to cover for possible cellulitis over his legs. His creatinine kinase levels slowly trended down. He remained persistently anuric during his admission. An ultrasound study of his kidneys and renal tract revealed normal kidneys with no hydronephrosis or obstruction and the rest of the renal tract was of normal description. A CT chest, abdomen, and pelvis was done to investigate for paraneoplastic causes of rhabdomyolysis, the outcome of this test was also unremarkable. A genetic panel study for the mitochondrial disease was sent following consultation with the mitochondrial disease specialist center, results were not available at the time of this publication.

Myositis, vasculitis, autoimmune, and virology screens were completed and the results were all negative. They included anti-Jo-1, anti-PL-7, anti-PL-12, anti-SRP (anti-signal recognition particle), anti-Ku, anti-Mi-2, anti-PM-Scl75, anti-PM-Scl100, anti-transcription intermediary factor 1-gamma (anti-TlF1 gamma), anti-melanoma differentiation-associated gene 5 (anti-MDA5), anti-nuclear matrix protein 2 (anti-NXP2), anti-SAE1, anti-Ro-52, anti-OJ, anti-EJ, anti-nuclear antibody, anti-double stranded DNA (anti-dsDNA) antibodies, anti-Ro(SSA), anti-La(SSB), anti-ribonucleoprotein antibody (anti-RNP), anti-smooth muscle antibody, anti-SCL-70 antibody, anti-neutrophil cytoplasmic antibody (ANCA), Rhesus (Rh) factor, Epstein-Barr virus (EBV) IgM, anti-HIV IgG and IgM, and HMGCo-A reductase antibody.

He improved to full sensorium while receiving care in the intensive care unit. He was then transferred to a regional renal center on day six of his intensive care unit admission for possible consideration of long-term dialysis and further investigations. He, however, received five sessions of intermittent dialysis and monitoring of his renal function at the renal center over a two-week period before he was eventually discharged home with no further need for renal replacement therapy. He was planned for further outpatient clinic follow-up visits by the orthopedic, renal, and genetic disorder units.

## Discussion

We have shared the case of a man with suspected m.9176T>C (ATP6) mitochondrial pathogenic variant (who had a positive family history for this genetic mutation) and had Charcot-Marie-Tooth disease-like peripheral neuropathy, who presented with two episodes of rhabdomyolysis over a two-year period and both episodes were related to periods around surgical interventions offered for patient's lower limb deformities. This case is unique in that it explores the possibility of delayed rhabdomyolysis as noted following the patient's second surgery in the context of the above mutation and its manifestations. We, therefore, highlight the importance of considering rhabdomyolysis as an important complication in the immediate and wider peri-operative period for this category of patients.

Mitochondrial diseases refer to a group of disorders that can generally be categorized as respiratory chain defects or fatty acid metabolism defects [4]. There is commonly multi-system involvement. They can cause a wide range of physiological changes which can have implications during the peri-operative period which clinicians should be aware of. The outcome of genetic studies done on the patient under review would have added more significance to this report, but the results were yet unavailable at the time of writing this report.

The m.9176T>C (ATP6) mitochondrial pathogenic variant is a mitochondrial DNA mutation and has a maternal inheritance pattern. It has been associated with the following syndromes: NARP syndrome (neuropathy, ataxia, and retinitis pigmentosa) and maternally-inherited Leigh syndrome (MILS) - an uncommon disorder that manifests with subacute necrotizing encephalopathy. m.9176 T>C (ATP 6) mutation has also been associated with Charcot-Marie-Tooth (CMT) disease-like pure peripheral neuropathy, which the patient under review has as part of his disease manifestation [5]. The patient developed lower limb deformities bilaterally and required orthopedic interventions (osteotomies) twice over a two-year period.

The m.9176T>C substitution causes an MT-ATP6 pathogenic, loss-of-function missense variant which codes for a subunit of the Fo ATP-synthase complex in complex V of the electron transport chain (ETC). Eight pathogenic variants have been described in ATP6 mutation [6]. The manifestations of ATP6 gene mutations vary, as noted in the above paragraph. The patient's neuropathy was assessed as being Charcot-Marie-Tooth disease-like with both sensory and motor function affectation. In comparison, the suspected m.9176T>C (ATP6) mitochondrial pathogenic variant in patients (with attendant CMT-like pure peripheral neuropathy) has a maternal inheritance pattern to it while typical Charcot-Marie-Tooth disease has been described to mainly have autosomal dominant, autosomal recessive or X-linked transmission patterns [7].

When possible, a thorough pre-operative anesthetics assessment should be conducted for patients with confirmed or suspected mitochondrial disease [3]. In particular, clinicians should familiarize themselves with the manifestations of the patient’s illness and be prepared for emergencies and complications that may arise from them. For instance, patients with POLG-related epilepsy are at a higher risk of morbidity and mortality due to seizures and their case should be discussed promptly with a mitochondrial disease specialist. Cardiomyopathy (dilated, hypertrophic, or mixed) can complicate mitochondrial disorders as is being reported in the cases of mitochondrial encephalopathy and lactic acidosis with stroke-like symptoms (MELAS) [8]. Furthermore, measures should be taken place to reduce metabolic stress in patients with mitochondrial disease. Practically, they should be considered to be first on the surgery list with a minimal fasting period. Following pre-assessment, clinicians should consider arranging close post-operative monitoring in a high-dependency unit or intensive care unit if deemed appropriate.

In terms of anesthetic agents used for patients with mitochondrial disease, there is no good evidence to suggest any anesthetic agents are superior to the others [3]. There is also no evidence to suggest that there are any increased risks specifically associated with anesthetics in this cohort of patients [9]. Although no anesthetic agents are contraindicated, special considerations should be taken due to the theoretical risk of some of them.

Propofol is one of the most common parenteral anesthetic agents used. It is known to interfere with mitochondrial metabolism by at least four different pathways. Given its multiple mitochondrial effects, it is recommended not to use any propofol-based anesthesia such as total intravenous anesthesia to reduce the risks on patients with mitochondrial disease. If hypotonia and mitochondrial myopathy are part of the patient’s disease manifestation, they would benefit from opioid-sparing methods such as local or regional anesthesia whenever possible. Avoidance of non-depolarizing neuromuscular blocking drugs is vital as they may exaggerate sensitivity [10].

The patient under review had both orthopedic procedures under general anesthesia. The first surgery was done aided with the use of a tourniquet to reduce bleeding during the osteotomy. The immediate post-operative period was complicated by the onset of rhabdomyolysis with swelling and tenderness affecting the operated limb. The patient required hemodiafiltration in the intensive care unit to manage the ensuing rhabdomyolysis. The second surgery was conducted without the use of a tourniquet and there was a delayed rhabdomyolysis three weeks after the surgery. Perhaps, the use of tourniquets should be rather avoided or used for limited times in orthopedic surgeries in patients with mitochondrial disorders.

In the post-operative period, patients with mitochondrial disease should receive routine care with special attention to hydration and calorific intake. Although there is scarce evidence to suggest an increased risk of rhabdomyolysis relating to surgery in adult mitochondrial disease, we think it may be vital for patients with mitochondrial disease to receive specific post-operative advice, given the case we have presented [3]. It is pertinent to inform patients of the importance of hydration and mobility in the post-operative period and more importantly to look out for signs of rhabdomyolysis, such as muscle pain and dark urine. They should escalate to their clinicians as early as possible if these signs arise. Clinicians should take note of any complications that have taken place during the peri-operative period and make necessary adjustments if further anesthesia or surgery is required.

## Conclusions

We have described a rare occurrence of rhabdomyolysis complicating two peri-operative periods for orthopedic interventions in a middle-aged male patient with a clinically suspected pathogenic mitochondrial DNA variation (likely in the form of m.9176T>C {ATP6} mitochondrial pathogenic variant) and Charcot-Marie-Tooth disease-like peripheral neuropathy. The patient has a positive family history of a confirmed diagnosis of the aforementioned genetic variant in his mother, sister, and maternal uncle. We have highlighted an earlier onset of rhabdomyolysis complicating the first orthopedic intervention that involved tourniquet use to limit blood loss. It would appear that the impact of rhabdomyolysis on patients with this syndrome may be worse than in normal individuals. More research studies are suggested in this direction. Multi-disciplinary team approach and adequate work-ups are suggested in the care of this rare group of patients presenting for surgeries.
